# Association of administration of IFN-α with mortality among patients hospitalized with coronavirus disease 2019

**DOI:** 10.2217/fvl-2020-0404

**Published:** 2021-03-03

**Authors:** Hanqing Liu, Zhouru Ruan, Ziwei Yin, Dan Wu, Hong Zhu

**Affiliations:** 1 ^1^Department of General Practice, The Central Hospital of Wuhan, Tongji Medical College, Huazhong University of Science & Technology, Wuhan, Hubei 430014, China; 2 ^2^Clinical College of Traditional Chinese Medicine, Hubei University of Chinese Medicine, Wuhan, Hubei 430065, China; 3 ^3^Department of Pharmacy, The Central Hospital of Wuhan, Tongji Medical College, Huazhong University of Science & Technology, Wuhan, Hubei 430014, China; 4 ^4^School of Medicine, Jianghan University, Wuhan, Hubei 430056, China; 5 ^5^Department of Teaching, The Central Hospital of Wuhan, Tongji Medical College, Huazhong University of Science & Technology, Wuhan, Hubei 430014, China

**Keywords:** administration, clinical characteristics, COVID-19, interferon, logistics analysis, mortality, SARS-CoV-2, survival curve

## Abstract

**Aim:** Recent studies on coronavirus disease 2019 (COVID-19) have not offered sufficient clinical evidence to support whether IFN-α can decrease the mortality of patients with COVID-19. **Method:** In this retrospective study, 103 of 1555 hospitalized COVID-19 patients were treated with IFN-α, and the others matched through propensity score matching. Cox regression model, logistics analysis and Kaplan–Meier statistics depicted the survival curve. **Results & conclusion: **Single factor analysis demonstrated that fewer deaths occurred in patients treated with IFN-α compared with patients treated without IFN-α (p = 0.000). Logistics analysis showed that patients treated with IFN-α had an all-cause mortality odds ratio = 0.01 (95% CI: 0.001–0.110; p = 0.000). The Cox regression model was utilized to determine an all-cause mortality with a hazard ratio of 0.102 (95% CI: 0.030–0.351; p = 0.000). IFN-α can alleviate disease severity and decrease all-cause mortality, especially in critical patients. IFN-α could effectively treat patients with COVID-19.

Coronavirus disease 2019 (COVID-19) broke out suddenly in 2019 and spread quickly across the world, affecting millionss of people. No specific medicine for COVID-19 has been found. The current study revealed that SARS coronavirus 2 (SARS-CoV-2), which is a +ssRNA and belongs to β-coronavirus [1], had a high consistency of 86.9% with the nucleic acid sequence of SARS coronavirus 1 (SARS-CoV-1). It is highly infectious, and the population is generally susceptible. Interferon (IFN) has been used in the past to treat patients with SARS-CoV-1 and Middle East respiratory syndrome coronavirus (MERS-CoV). Studies have shown that IFN is one of the first cytokines produced after SARS-CoV-2 infects the host, and it can participate in the regulation of inflammation and immunity. Thus far, there are few reports on the effect of IFN-α on COVID-19 [2]. The latest version of the Chinese Diagnosis and Treatment Protocol for Novel Coronavirus Pneumonia continues to recommend injections and nebulizers for IFN-α treatment [3], but current studies show that IFN may exacerbate the inflammatory response in the treatment of COVID-19, which have contradictory conclusions on the role of IFN-α [4,5]. Therefore, this article will mainly explore whether administration of IFN-α can decrease the mortality of hospitalized patients with COVID-19.

## Methods

This is a retrospective study that includes 1555 patients who visited The Central Hospital of Wuhan from December 30, 2019, to April 30, 2020, and were diagnosed with COVID-19 based on the Chinese Diagnosis and Treatment Protocol for Novel Coronavirus Pneumonia [3]. Recombinant human IFN-α1b for injection (Shenzhen Kexing Biotech Co., Ltd Shenzhen, China) and recombinant human IFN-α2b spray (Tianjin Sinbobioway Biology, Tianjin, China) were used in this study. IFN-α1b 50 ug was added to 1 ml of sterile water and administered as an intramuscular injection once a day. IFN-α2b 5 mIU was sprayed into the nose two-times a day for a total of 10 mIU per day. The severity of COVID-19 was defined based on the Chinese Diagnosis and Treatment Protocol for Novel Coronavirus Pneumonia, and patients were divided into four types: mild, moderate, severe and critical. In this study, patients with the mild or moderate type were defined as mild, and the definitions of severe and critical were retained; thus, the population was divided into three groups. Severe cases met any of the following criteria: respiratory distress (≥30 breaths/min), oxygen saturation ≤93% at rest or PaO2/FiO2 ≤300 mmHg (1 mmHg = 0.133 kPa). In high-altitude areas (>1000 m above sea level), PaO2/FiO2 was corrected using the following formula: PaO2/FiO2 × [atmospheric pressure (mmHg)/760]. Cases with chest imaging that showed lesion progression >50% within 24–48 h were managed as severe cases. Critical cases met any of the following criteria: respiratory failure requiring mechanical ventilation, shock or other organ failure that required intensive care unit care.

## Statistical analysis

In this study, propensity score matching was performed based on the incidence of potential confounding factors related to the administration of IFN-α, including age. Based on the missForest program in the R Project for Statistical Computing, nonparametric missing value estimation was adopted to explain missing data on laboratory variables, such as IL-6, C-reactive protein, procalcitonin and D-dimer. The remaining variables in the data were used to perform a random forest model to predict the values of the missing variables. Patients treated with IFN-α (IFN group) and those not treated with IFN-α (non-IFN group) were matched according to propensity score matching, and the exact matching caliper size was 0.01. The balance of covariates was evaluated by estimating the standard deviation before and after matching. When the absolute value was less than 0.1, the balance between the two groups was considered to be successful. The matching ratio of IFN group to non-IFN group was 1:1.

Continuous variables were represented by median and interquartile range, and categorical variables were represented by quantity and percentage. The Mann–Whitney U test was used for continuous variables to analyze the statistical differences between the two groups, and the Fisher exact test or chi-square test was used for categorical variables for comparison. To explore the interactions among risk factors in COVID-19 patients treated with IFN-α, a binary logistic regression model was used to estimate odds ratio (OR) and 95% CI, making adjustments based on factors such as age, sex, symptoms, signs and complications. The Cox regression model was used to compare the IFN group and the non-IFN group and calculated the corresponding hazard ratio and 95% CI of the disease outcome. Considering patient death as a terminal event, the Kaplan–Meier method was used to compare the cumulative survival rate. Bilateral lateral position <0.01 was considered a significant statistical difference, <0.05 was considered a statistical difference and p > 0.05 was considered no statistical difference. Data analysis was performed using R-3.6.3 (R Project for Statistical Computing, Vienna, Austria) and SPSS Statistics 22.0 (IBM, NY, USA) [6].

## Results

As of April 30, 2020, a total of 1555 patients were hospitalized for COVID-19. The median patient age was 57 years old, and 723 were male (46.5%), with no significant difference in age or sex. The median length of hospital stay was 17 days. There were 653 (42.0%) mild cases, 600 severe cases (38.6%) and 302 (19.4%) critical cases. A total of 140 patients did not survive (9.0% mortality). At first admission, the primary symptom was chest tightness (33.9%), followed by nausea (30.6%), fever (18.5%), cough (11.3%), headache (10.6%), chest pain (5.6%), fatigue (4.6%), dyspnea (2.5%) and diarrhea (2.3%) (Table 1).

**Table 1.** Characteristics of IFN group compared with non-IFN group in patients with COVID-19.

		Total, n = 206	IFN, n = 103	Non-IFN, n = 103	p-value
Age, median, years (IQR)		51 (36–66)	49 (36–64)	55 (37–66)	0.481
Sex (%)					
Male		102 (49.5)	54 (52.4)	48 (46.6)	0.403
Female		104 (50.5)	49 (47.6)	55 (53.4)	
Nonsurvivor (%)		22 (10.7)	3 (2.9)	19 (18.4)	0.000
Hospital stay, median, days (IQR)		18.5 (8.0–26.3)	22.0 (16.0–30.0)	9.00 (4.0–22.0)	0.000
**Severity (%)**					
Mild		65 (31.6)	33 (32.0)	32 (31.1)	0.012
Severe		94 (45.6)	55 (53.4)	39 (37.9)	
Critical		47 (22.8)	15 (14.6)	32 (31.1)	
**Laboratory data, median (IQR)**	Normal range				
Leukocytes, ×10^9^ cells/l	3.5–9.5	4.6 (3.4–5.9)	4.5 (3.4–5.8)	4.6 (3.4–6.0)	0.726
Neutrophils, ×10^9^ cells/l	1.8–6.3	2.9 (2.0–4.2)	2.9 (2.0–4.0)	3.0 (2.0–4.3)	0.880
Lymphocytes, ×10^9^ cells/l	1.1–3.2	1.1 (0.7–1.40)	1.0 (0.8–1.4)	1.1 (0.7–1.4)	0.979
Monocytes, ×10^9^ cells/l	0.1–0.6	0.3 (0.2–0.5)	0.3 (0.2–0.4)	0.4 (0.2–0.6)	0.006
Eosinophils, ×10^9^ cells/l	0.02–0.52	0.01 (0.00–0.04)	0.00–0.04	0.01 (0.00–0.02)	0.669
Basophils, ×10^9^ cells/l	<0.06	0.01 (0.01–0.02)	0.01 (0.01–0.02)	0.01 (0.01–0.02)	0.736
Lymphocytes, %	20–50	24.5 (15.9–33.7)	24.3 (16.9–34.6)	25.0 (15.3–33.4)	0.965
Neutrophils, %	40–75	64.6 (56.6–76.7)	64.5 (57.1–76.9)	64.6 (53.5–76.7)	0.424
Monocytes, %	3–10	7.6 (5.4–9.9)	6.8 (4.7–9.2)	8.2 (6.0–10.7)	0.002
Eosinophils, %	0.4–8.0	0.2 (0.0–0.7)	0.20 (0.0–0.8)	0.1 (0.0–0.6)	0.593
Basophils, %	<1	0.2 (0.1–0.4)	0.2 (0.1–0.4)	0.2 (0.1–0.4)	0.951
Platelets, ×10^9^ cells/l	125–350	173 (134–212)	173 (135–217)	172 (132–209)	0.469
Hemoglobin, ×10^9^ g/l	130–175	131 (122–141)	131 (122–141)	129 (117–140)	0.314
Activated partial thromboplastin time, s	20–40	27.0 (24.4–30.1)	28.8 (25.6–32.0)	25.3 (23.5–28.2)	0.000
Fibrinogen, g/l	2–4	3.00 (2.46–3.64)	2.75 (2.29–3.12)	3.34 (2.79–4.09)	0.000
Prothrombin time, s	9–13	11.2 (10.7–11.7)	11.3 (11.0–12.0)	11.0 (10.5–11.6)	0.000
International normalized ratio	0.7–1.3	0.96 (0.91–1.01)	0.97 (0.95–1.04)	0.93 (0.89–0.99)	0.000
D-dimer, ug/ml	<1	0.4 (0.2–1.0)	0.4 (0.2–0.9)	0.4 (0.2–1.1)	0.807
Albumin, g/l	40–55	40.9 (37.0–43.5)	41.6 (38.1–43.7)	39.6 (36.0–42.6)	0.009
Globulin, g/l	20–40	27.6 (24.8–31.4)	26.7 (23.3–29.2)	29.3 (26.2–31.8)	0.000
Albumin-to-globulin ratio	1.2–2.4	1.5 (1.2–1.6)	1.6 (1.4–1.8)	1.3 (1.2–1.5)	0.000
Alanine aminotransferase, U/l	9–50	18.5 (13.0–29.5)	18.6 (12.8–28.2)	18.4 (13.1–33.0)	0.775
Aspartate aminotransferase, U/l	15–40	23.7 (17.2–35.6)	21.0 (16.5–31.0)	26.0 (19.0–36.6)	0.016
Total bilirubin, mmol/l	2.0–20.4	8.6 (6.4–11.8)	8.9 (7.2–11.6)	8.0 (5.8–11.9)	0.077
Calcium, mmol/l	2.2–2.7	2.4 (2.2–2.6)	2.2 (2.1–2.3)	2.6 (2.5–2.7)	0.000
Urea, mmol/l	1.7–8.3	4.2 (3.3–5.2)	4.0 (3.1–4.8)	4.5 (3.5–5.7)	0.002
Creatinine, umol/l	57–111	62.7 (52.8–76.3)	62.2 (51.6–75.2)	62.9 (54.9–81.0)	0.108
Alkaline phosphatase, U/l	40–150	52.6 (44.0–63.1)	49.3 (42.0–62.0)	54.9 (48.3–66.0)	0.002
Creatine kinase, U/l	<190	83.3 (48.8–145.1)	77.0 (48.0–121.0)	95.4 (50.3–159.0)	0.096
Lactate dehydrogenase, U/l	80–285	175.5 (140.5–229.0)	172.0 (142.0–212.0)	176.8 (137.0–238.0)	0.722
Angiotensin-converting enzyme, U/l	12–68	19.9 (17.3–23.8)	22.5 (19.8–26.3)	18.1 (15.0–19.9)	0.000
Myoglobin, ng/ml	<154.90	38.7 (25.5–69.4)	28.3 (19.4–50.4)	48.2 (33.7–87.7)	0.000
Troponin, ng/ml	<0.034	0.01 (0.00–0.01)	0.01 (0.00–0.01)	0.01 (0.00–0.01)	0.732
Blood glucose, mmol/l	3.9–6.1	5.8 (5.0–7.2)	5.7 (5.0–7.3)	5.94 (5.1–7.0)	0.564
Procalcitonin, ng/ml	<0.05	0.05 (0.04–0.08)	0.05 (0.04–0.08)	0.05 (0.02–0.08)	0.258
IL-6, pg/ml	0–7	13.6 (5.4–35.3)	5.4 (2.9–11.4)	30.5 (14.2–61.1)	0.000
Erythrocyte sedimentation rate, mm/h	<15	32.7 (22.0–45.5)	29.8 (21.0–44.3)	35.6 (22.7–47.1)	0.162
C-reactive protein, mg/dl	<0.6	1.8 (0.5–3.9)	1.6 (0.2–3.9)	1.8 (0.6–4.0)	0.289
Creatine kinase-myocardial band, U/l	24	8 (6–12)	7 (5–9)	10 (7–13)	0.000
Alpha hydroxybutyrate, U/l	72–182	137 (113–172)	140 (114–171)	136 (110–183)	0.935
γ-glutamyl transpeptidase, U/l	10–60	21.3 (13.3–39.0)	21.1 (13.0–36.0)	21.4 (13.9–41.9)	0.576
Brain natriuretic peptide, pg/ml	<100	59.4 (30.7–117.6)	61.1 (27.9–117.0)	56.9 (31.0–133.2)	0.706
**Treatment (%)**					
IFN		103 (50.00)	103 (100.00)	0 (0.00)	
IFN-α1b spray		15 (7.30)	15 (14.60)	0 (0.00)	0.000
IFN-α2b		89 (43.20)	89 (86.40)	0 (0.00)	0.000
Ribavirin		183 (88.80)	93 (90.30)	90 (87.40)	0.507
Arbidol		74 (35.90)	67 (65.00)	7 (6.80)	0.000
Oseltamivir		107 (51.90)	31 (30.10)	76 (73.80)	0.000
Ganciclovir		18 (8.70)	4 (3.90)	14 (13.60)	0.014
Lopinavir		0 (0.00)	0 (0.00)	0 (0.00)	
Antibiotics		192 (93.20)	91 (88.30)	101 (98.10)	0.006
Glucocorticoid		139 (67.50)	73 (70.90)	66 (64.10)	0.298
Antivirotic		198 (96.10)	101 (98.10)	97 (94.20)	0.279
Antifungal		15 (7.30)	3 (2.90)	12 (11.70)	0.016
Gamma globulin		63 (30.60)	50 (48.50)	13 (12.60)	0.000
Noninvasive positive pressure ventilation		32 (15.50)	13 (12.60)	19 (18.40)	0.248
Intermittent positive pressure ventilation		25 (12.10)	6 (5.80)	19 (18.40)	0.006
**Clinical symptoms (%)**					
Fever		33 (16.00)	14 (13.60)	19 (18.40)	0.342
Cough		29 (14.10)	9 (8.70)	20 (19.40)	0.028
Dyspnea		10 (4.90)	3 (2.90)	7 (6.80)	0.195
Diarrhea		6 (2.90)	0 (0.00)	6 (5.80)	0.029
Headache		28 (13.60)	8 (7.80)	20 (19.40)	0.015
Chest pain		12 (5.80)	2 (1.90)	10 (9.70)	0.017
Nausea		94 (45.60)	43 (41.70)	51 (49.50)	0.263
Fatigue		11 (5.30)	2 (1.90)	9 (8.70)	0.030
Chest distress		53 (25.70)	19 (18.40)	34 (33.00)	0.017
**Chronic disease and complications (%)**					
Diabetes mellitus		2 (1.00)	1 (1.00)	1 (1.00)	1.000
Coronary artery disease		3 (1.50)	1 (1.00)	2 (1.90)	1.000
Chronic obstructive pulmonary disease		43 (20.90)	23 (22.30)	20 (19.40)	0.607
Heart failure		132 (64.10)	76 (73.80)	56 (54.40)	0.004
Cerebrovascular disease		108 (52.40)	75 (72.80)	33 (32.00)	0.000
Chronic kidney disease		29 (14.10)	20 (19.40)	9 (8.70)	0.028
Digestive system disease		13 (6.30)	8 (7.80)	5 (4.90)	0.390
Cancer		20 (9.70)	8 (7.80)	12 (11.70)	0.347
Hypertension		8 (3.90)	2 (1.90)	6 (5.80)	0.279
Viral hepatitis		9 (4.40)	4 (3.90)	5 (4.90)	1.000
Acute respiratory distress syndrome		41 (19.90)	14 (13.60)	27 (26.20)	0.023
Septic shock		34 (16.50)	19 (18.40)	15 (14.60)	0.453
Acute liver injury		37 (18.00)	24 (23.30)	13 (12.60)	0.046
**Lung CT (%)**					
Bilateral lesions		147 (71.40)	84 (81.60)	63 (61.20)	0.001
Patchy shadow		138 (67.00)	83 (80.60)	55 (53.40)	0.000
No lesions		29 (14.10)	4 (3.90)	25 (24.30)	0.000

COVID-19: Coronavirus disease 2019; CT: Computed tomography; IFN: Interferon; IQR: Interquartile range.

After the analysis of the past history of the patients, it was found that there were 20 with diabetes (1.3%), 23 with coronary artery disease (1.5%), 214 with chronic obstructive pulmonary disease (13.8%), 966 with heart failure (62.1%), 727 with cerebrovascular disease (46.8%), 216 with kidney disease (13.9%), 81 with digestive system disease (5.2%), 69 with tumor (4.4%), 36 with hypertension (2.3%) and 79 with viral hepatitis (5.1%).

During the hospitalization, 248 patients (15.9%) suffered septic shock, 242 (15.6%) acute liver injury and 220 (14.1%) acute respiratory distress syndrome. Through further statistical analysis of the lung computed tomography (CT), no lesions was 242 (15.6%), patchy shadows 850 (54.7%) and bilateral lesions 994 (63.9%).

The characteristics of the clinical symptoms, lung CT, laboratory data, related complications and treatment of patients are summarized in Table 2. Here the authors mainly describe the relevant data after propensity score matching. Based on age as an important potential confounding factor of exposure in the IFN group, the authors obtained relevant data on the two groups after matching the propensity score 1:1. After comparing the IFN group and non-IFN group, it was found that there was no statistical difference in gender (p = 0.481). Exploring the effect of the administration of IFN-α, it was found that the number of nonsurvivors in the IFN was lower than that in the non-IFN group (p = 0.000), so as well as the length of hospital stay (p = 0.000). In addition, the disease severity was statistically different from the treatment with IFN-α (p = 0.012).

Patients' clinical symptoms, lung CT and laboratory data at the time of admission were collected. It was found that clinical symptoms such as cough, diarrhea, headache, chest pain, fatigue and chest tightness were statistically different during hospitalization (p < 0.05). Monocyte count and percentage, activated partial thromboplastin time, fibrinogen, prothrombin time, international normalized ratio, albumin, globulin, albumin-to-globulin ratio, urea, alkaline phosphatase, angiotensin-converting enzyme (ACE), myoglobin, IL-6, creatine kinase-myocardial band and calcium had a significant statistical difference (p < 0.01), as did aspartate aminotransferase (p < 0.05). Lung CT statistics were divided into no lesions, patchy shadows and bilateral lesions, and the results showed that the difference was significant between the IFN group and the non-IFN group with respect to no lesions, patchy shadows and bilateral lesions (p < 0.01).

Through the comparison of differences between the IFN group and the non-IFN group after treatment with antibiotics, antiviral drugs, glucocorticoids, antifungal drugs and gamma globulin, it was found that treatment with antibiotics (p = 0.046), gamma globulin (p = 0.000), arbidol (p = 0.000) and oseltamivir (p = 0.000) showed statistically significant differences between the two groups, whereas treatment with glucocorticoids showed no statistically significant difference (p = 0.298). Further analysis found that there was a statistically significant difference between the two groups in patients treated with intermittent positive pressure ventilation (p = 0.006), whereas there was no statistical difference in patients treated with noninvasive positive pressure ventilation (p = 0.248).

The authors hypothesized that the presence of comorbid conditions was related to morbidity, and it was found that there was a significant statistical difference in survival between patients with no comorbid conditions who were treated with IFN and patients with previous heart failure and cerebrovascular disease (p < 0.01). Moreover, survival rate in patients with chronic kidney disease was statistically different (p = 0.028), whereas survival rate in patients with diabetes, hypertension and chronic obstructive pulmonary disease showed no statistical difference (p > 0.05). Finally, the authors regarded septic shock, acute liver injury and acute respiratory distress syndrome as representative of complications in COVID-19 patients, and the results showed that acute liver injury and acute respiratory distress syndrome were statistically different in the IFN group (p < 0.05), whereas there was no statistical difference in septic shock (p = 0.453).

Analyzing the relationship between treatment with IFN-α and disease severity, the authors found no association between administration of IFN-α and mortality in patients with mild and severe cases (p > 0.05), whereas a statistical difference was seen between administration of IFN-α and mortality in patients with critical cases (p = 0.028). In terms of complications, there was a statistical difference between treatment with IFN-α and the occurrence of acute liver injury in patients with severe cases (p = 0.031), whereas no statistically significant difference was seen between treatment with IFN-α and the occurrence of acute liver injury in patients with mild and critical cases (p > 0.05). Compared with patients not treated with IFN-α, there was no difference between treatment with IFN-α1b spray and the severity of disease (p = 0.419); however, there was a statistical difference between treatment with IFN-α2b injection and disease severity (p = 0.011) (Table 2).

**Table 2.** Characteristics and severity of IFN group compared with non-IFN group in patients with COVID-19.

Characteristic	Mild			Severe			Critical		
	IFN, n = 33	Non-IFN, n = 32	p-value	IFN, n = 55	Non-IFN, n = 39	p-value	IFN, n = 15	Non-IFN, n = 32	p-value
Age, median, years (IQR)	37 (33–53)	36 (32–46)	0.541	59 (38–67)	56 (37–72)	0.875	57 (39–69)	64 (56–75)	0.100
Sex (%)									
Male	13 (39.40)	13 (40.60)	1.000	30 (54.50)	19 (48.70)	0.676	11 (73.30)	16 (50.00)	0.206
Female	20 (60.60)	19 (59.40)		25 (45.50)	20 (51.30)		4 (26.70)	16 (50.00)	
Nonsurvivor (%)	0 (0.00)	0 (0.00)	-	0 (0.00)	0 (0.00)	-	3 (20.00)	18 (56.30)	0.028
Hospital stay, median, days (IQR)	19.0 (14.0–23.5)	4 (3.0–6.8)	0.000	23.0 (19.0–35.0)	9.00 (6.0–16.0)	0.000	29.0 (15.0–49.0)	24.5 (17.5–43.8)	0.479
**Chronic disease and complications (%)**									
Diabetes mellitus	0 (0.00)	0 (0.00)	-	1 (1.80)	0 (0.00)	1.000	1 (3.10)	0 (0.00)	1.000
Coronary artery disease	0 (0.00)	0 (0.00)	-	0 (0.00)	0 (0.00)	-	1 (6.70)	2 (6.30)	1.000
Chronic obstructive pulmonary disease	4 (12.10)	4 (12.50)	1.000	12 (21.80)	6 (15.40)	0.596	7 (46.70)	10 (31.30)	0.344
Heart failure	23 (69.70)	23 (71.90)	1.000	44 (80.00)	21 (53.80)	0.012	9 (60.00)	12 (37.50)	0.211
Cerebrovascular disease	10 (75.80)	25 (31.30)	0.000	43 (78.20)	14 (35.90)	0.000	7 (46.70)	9 (28.10)	0.322
Chronic kidney disease	2 (6.10)	0 (0.00)	0.492	16 (29.10)	2 (5.10)	0.007	2 (13.30)	7 (21.90)	0.697
Digestive system disease	1 (3.00)	1 (3.10)	1.000	4 (7.30)	1 (2.60)	0.339	3 (20.00)	3 (9.40)	0.367
Cancer	6 (18.20)	5 (15.60)	1.000	1 (1.80)	6 (15.40)	0.019	1 (6.70)	1 (3.10)	0.541
Hypertension	0 (0.00)	1 (3.10)	0.492	2 (3.60)	3 (7.70)	0.646	0 (0.00)	2 (6.30)	1.000
Viral hepatitis	0 (0.00)	1 (3.10)	0.492	2 (3.60)	3 (7.70)	0.646	2 (13.30)	1 (3.10)	0.235
Acute respiratory distress syndrome	0 (0.00)	0 (0.00)	-	0 (0.00)	0 (0.00)	-	14 (93.30)	27 (84.40)	0.648
Septic shock	5 (15.20)	5 (15.60)	1.000	8 (14.50)	8 (20.50)	0.579	6 (40.00)	2 (6.30)	0.009
Acute liver injury	7 (21.20)	3 (9.40)	0.303	15 (27.30)	3 (7.70)	0.031	2 (13.30)	7 (21.90)	0.697
**IFN (%)**									
IFN-α1b spray	7 (10.8)	0 (0.00)	-	5 (5.3)	0 (0.00)	-	3 (6.4)	0 (0.00)	0.419^†^
IFN-α2b	26 (40)	0 (0.00)	-	50 (53.2)	0 (0.00)	-	13 (27.7)	0 (0.00)	0.011^‡^

† The p-value shows the relationship between treatment with IFN-α1b spray and disease severity compared with patients not treated with IFN.

‡ The p-value shows the relationship between treatment with IFN-α2b injection and disease severity compared with patients not treated with IFN.

COVID-19: Coronavirus disease 2019; IFN: Interferon; IQR: Interquartile range.

Through logistic regression analysis, it was found that patients treated with IFN-α had an all-cause mortality OR = 0.01 (95% CI: 0.001–0.11; p = 0.000), fibrinogen OR = 0.231 (95% CI: 0.085–0.627; p = 0.004) and albumin OR = 1.294 (95% CI: 1.095–1.530; p = 0.002). Compared with the non-IFN group, the IFN group had a preexisting coronary artery disease OR = 0.018 (95% CI: 0.001–0.617; p = 0.026). For patients treated with IFN-α, the OR value of arbidol treatment was 19.613 (95% CI: 3.681–104.515; p = 0.000) (Table 3).

**Table 3.** Results of IFN group and non-IFN group logistic regression analysis in patients with COVID-19.

	B	SE	Wald	p-value	OR	95% CI	
						Lower	Upper
Nonsurvivor	-4.647	1.244	13.964	0.000	0.010	0.001	0.110
Fibrinogen	-1.467	0.511	8.258	0.004	0.231	0.085	0.627
Albumin	0.258	0.085	9.160	0.002	1.294	1.095	1.530
Calcium	-17.108	3.279	27.225	0.000	0.000	0.000	0.000
Arbidol	2.976	0.854	12.155	0.000	19.613	3.681	104.515
Coronary artery disease	-4.039	1.815	4.955	0.026	0.018	0.001	0.617

COVID-19: Coronavirus disease 2019; IFN: Interferon; SE: Standard error.

**Figure 1.**
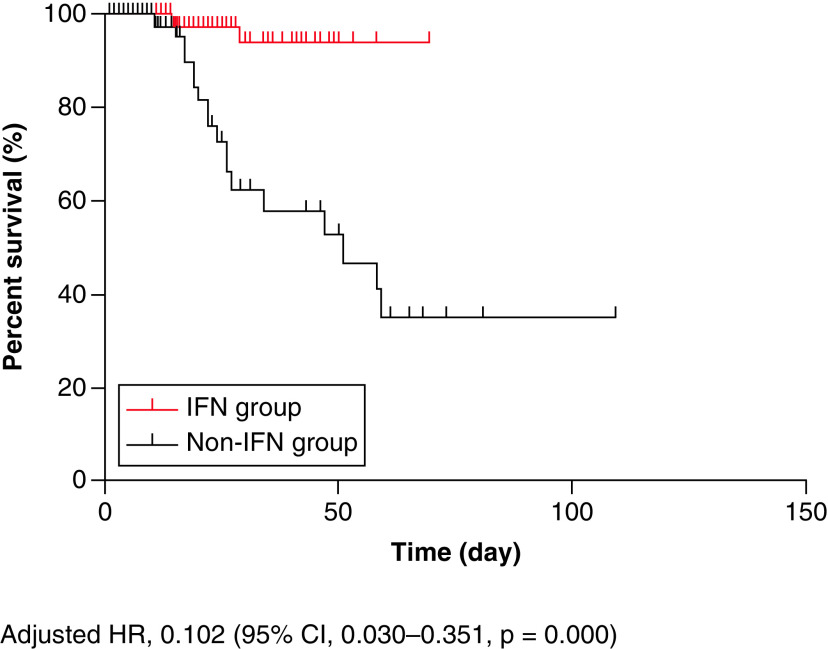
Survival analysis of the IFN group and the non-IFN group in patients with COVID-19. IFN: Interferon.

The survival curve was depicted by Kaplan–Meier statistics, and the Cox proportional hazard model was used to describe the 0.102 all-cause mortality hazard ratio (95% CI: 0.03–0.351; p = 0.000) in the IFN group and the non-IFN group (Figure 1).

## Discussion

In this study, the data the authors report show that patients hospitalized with COVID-19 and treated with IFN-α have a lower all-cause mortality compared with patients not treated with IFN-α. Furthermore, through the analysis of the correlation between treatment with IFN-α and the severity of disease, it was found that IFN-α can alleviate disease severity in patients with COVID-19 and decrease mortality in critically ill patients. Therefore, the authors hold that it is feasible to treat COVID-19 patients with appropriate administration of IFN-α.

The IFN system is composed of a series of antiviral IFN cytokines. On the basis of different molecular characteristics and recognition receptors in cells, these can be classified as type I, II and III and can induce a variety of IFN-stimulated effector genes and perform a variety of antiviral and other immunomodulatory functions. IFN-I is further divided into different subtypes, and IFN-α is one of the common subtypes produced by most cells [1]. Existing research confirms that SARS-CoV-2 first infects type I and II alveolar epithelial cells and alveolar macrophages through ACE2. IFN-I is one of the first cytokines produced after SARS-CoV-2 infects the host. By inhibiting early viral replication, limiting the spread of SARS-CoV-2 in the host [7], IFN is essential for regulating the activation and function of various immune cell groups as the central link between the innate immune system and the adaptive immune system [8]. Currently, it is believed that SARS-CoV-2 can inhibit the host's innate immunity, unbalance innate and acquired immune responses and induce low-level expression of IFN and pro-inflammatory cytokines/chemokines to delay virus clearance. IFN can also be used in the peripheral blood of severely ill COVID-19 patients. Trace amounts of IFN have been detected in the peripheral blood and lungs of severe COVID-19 patients. The low-level expression of IFN is related to severe cases [4], which provides theoretical support for the use of IFN in the treatment of COVID-19.

The antagonistic mechanism of SARS-CoV-2 is similar to that of other severe human coronaviruses, such as SARS-CoV and MERS-CoV, which can interfere with the host IFN signaling pathway, especially the production of IFN-I [1]. Moreover, IFN has been used in the treatment of SARS and MERS. It has been found through analogy study of IFN in SARS-CoV-1 and MERS-CoV that IFN can upregulate the expression of ACE2 [9], yet the correlation between ACE2 expression and COVID-19 is controversial. The upregulation of ACE2 expression increases the corresponding protective effect, strengthens the anti-inflammatory effect of ACE2 and weakens the damaging effect of angiotensin II. However, based on the molecular mechanism of ACE2 combined with host TMPRSS2 and other cathepsin-mediating virus invasion into target cells, some scholars believe that ACE2 may be a new type of IFN-stimulated effector gene induced by IFN and RNA sensing pathways, promoting the entrance of SARS-CoV-2 into the host cell. The induction of ACE2 by inflammatory cytokines also means that the ‘cytokine storm' caused by SARS-CoV-2 not only damages host tissues but may also accelerate the spread of the virus [10].

Recently, Acharya *et al.* postulated that dysregulated IFN is the key to the pathogenesis of COVID-19 [11]. Both early deficiency and late persistent high levels of IFN-I may be the key to the severity of COVID-19 [4]. Jeong *et al.* claim that the timing of IFN-I is a key factor in determining the outcome of infection [12]. Delayed IFN-I response leads to pathological inflammation, whereas early treatment with IFN-I can control viral replication. Park *et al.* believe that early administration of IFN-I or preventive treatment can provide the greatest degree of protection before the viral peak [13]. Treatment with IFN-α can limit the replication of the virus in the upper respiratory tract and decrease the spread of the virus in the lungs. In the later stages of the disease, IFN should be utilized with caution to avoid exacerbating inflammation and tissue damage. This has also been proven in mouse models of SARS-CoV-1 and MERS-CoV [14,15]. Whether IFN-α has a protective or harmful effect on the host may be dependent on the stage of infection and related factors. Consistent with the results of the current study, therefore, the authors infer that this may be closely related to IFN-α inhibiting early replication of the virus, indirectly enhancing the effect of antiviral drugs, and IFN signaling stimulating the establishment of the immune response, which plays a role in immune regulation. This further illustrates the importance of early initiation of IFN therapy. Therefore, the authors hold that better treatment effect can be achieved if patients with COVID-19 are treated with IFN-α earlier.

In this study, the authors found that treatment with IFN combined with arbidol, with an OR value of 19.613, may increase the clinical benefit in COVID-19 patients. Similarly, Zhou *et al.* found that the combination therapy of lopinavir/ritonavir, IFN-α and arbidol shortened the shedding time of SARS-CoV-2 and was considered to be an independent factor related to the duration of virus shedding [16]. Zuo *et al.* looked at 77 SARS-CoV-2 patients administered IFN-α2b in aerosolized form in a retrospective study that compared the efficacy of aerosolized IFN-α2b or umifenovir monotherapy with IFN-α2b combined with umifenovir, the result of which showed that patients treated with IFN-α cleared the virus faster than patients treated with umifenovir alone [17]. This is consistent with the case shared by Xie *et al.* involving lopinavir/ritonavir combined with arbidol and IFN-α1b combined with antiviral therapy [18]. Therefore, arbidol is preferred when combined antiviral therapy is necessary for patients already treated with IFN.

Currently, some studies show that SARS-CoV-2 can also overstimulate macrophages and neutrophils and continuously activate the immune system, which can lead to blood phagocytic syndrome, uncontrolled expansion of cytokine production and inflammatory storm [19,20]. Once macrophage activation syndrome/hemophagocytic lymphohistiocytosis occurs, antiviral therapy alone is not enough, and appropriate anti-inflammatory therapy should be added. In this study, the results demonstrate that antibiotics (p = 0.046) are statistically different between the two groups. In addition, monocytes of the two groups are significantly different (p < 0.01), which is consistent with the study of virus tracking technology that SARS-CoV-2 mainly infects epithelial and macrophage subgroups [21].

## Conclusion

The administration of IFN-α during hospitalization for COVID-19 can alleviate disease severity and decrease all-cause mortality, especially in critically ill patients. Patients with COVID-19 would be better treated by earlier administration of IFN-α. However, this is a retrospective study, and differences between patients in the IFN group and those in the non-IFN group at the time of admission may lead to bias. However, the baseline characteristics measured were similar in both groups (Table 1), indicating that the two groups were fairly comparable. Because timing of IFN could not be collected in this study, the authors plan to conduct further randomized controlled studies to clarify the therapeutic effect and timing of IFN in patients with COVID-19. Looking forward, further attempts could prove quite beneficial to the literature.

**Summary points**

- Patients hospitalized with coronavirus disease 2019 and treated with IFN-α have a lower all-cause mortality compared with patients not treated with IFN-α.
- IFN-α can alleviate disease severity in patients with coronavirus disease 2019 and decrease mortality in critically ill patients.
- Early deficiency and late persistent high levels of IFN-α may be the key to disease severity. Hence, earlier treatment with IFN-α would be more beneficial.
- Arbidol is preferred when combined antiviral therapy is necessary for patients already treated with IFN-α.

## References

[1] Lopez L, Sang PC, Yun T Dysregulated interferon response underlying severe COVID-19. Viruses 12(12), 1433 (2020). 10.3390/v12121433 PMC7764122 33322160

[2] Yang A, Guduguntla LS, Yang B. Potentials of interferons and hydroxychloroquine for the prophylaxis and early treatment of COVID-19. J. Cell Immunol. 2(6), 333–340 (2020). 33426541 10.33696/immunology.2.063 PMC7793568

[3] Wei PF. Diagnosis and treatment protocol for novel coronavirus pneumonia (trial version 7). Chin. Med. J. 133(9), 1087–1095 (2020). 32358325 10.1097/CM9.0000000000000819 PMC7213636

[4] Blanco Melo D, Nilsson Payant BE, Liu WC Imbalanced host response to SARS-CoV-2 drives development of COVID-19. Cell 181(5), 1036–1045 (2020). 32416070 10.1016/j.cell.2020.04.026 PMC7227586

[5] Hadjadj J, Yatim N, Barnabei L Impaired type I interferon activity and exacerbated inflammatory responses in severe COVID-19 patients. Science 369(6504), 718–724 (2020). 32661059 10.1126/science.abc6027 PMC7402632

[6] Peng Z, Lihua Z, Jingjing C. Association of inpatient use of angiotensin-converting enzyme inhibitors and angiotensin II receptor blockers with mortality among patients with hypertension hospitalized with COVID-19. Circ. Res. 126(12), 1671–1681 (2020). 32302265 10.1161/CIRCRESAHA.120.317134 PMC7265882

[7] Ludmila PO, Noémie A, Ruth ED COVID-19 and emerging viral infections: the case for interferon lambda. J. Exp. Med. 217(5), e20200653 (2020). 32289152 10.1084/jem.20200653 PMC7155807

[8] Crow MK, Ronnblom L. Type I interferons in host defence and inflammatory diseases. Lupus Sci. Med. 6(1), e000336 (2019). 31205729 10.1136/lupus-2019-000336 PMC6541752

[9] Ziegler CGK, Allon SJ, Nyquist SK SARS-CoV-2 receptor ACE2 is an interferon-stimulated gene in human airway epithelial cells and is detected in specific cell subsets across tissues. Cell 181(5), 1016–1035 (2020). 32413319 10.1016/j.cell.2020.04.035 PMC7252096

[10] Zhuang MW, Cheng Y, Zhang J Increasing host cellular receptor-angiotensin-converting enzyme 2 expression by coronavirus may facilitate 2019-nCoV (or SARS-CoV-2) infection. J. Med. Virol. 92(11), 2693–2701 (2020). 32497323 10.1002/jmv.26139 PMC7300907

[11] Acharya Dhiraj LG, Gack Michaela U. Dysregulation of type I interferon responses in COVID-19. Nat. Rev. Immunol. 20, 397–398 (2020). 32457522 10.1038/s41577-020-0346-x PMC7249038

[12] Jeong SL, Seongwan P, Hye WJ Immunophenotyping of COVID-19 and influenza highlights the role of type I interferons in development of severe COVID-19. Sci. Immunol. 5(49), eabd1554 (2020). 32651212 10.1126/sciimmunol.abd1554 PMC7402635

[13] Park A, Iwasaki A. Type I and type III interferons – induction, signaling, evasion, and application to combat COVID-19. Cell Host Microbe 27(6), 870–878 (2020). 32464097 10.1016/j.chom.2020.05.008 PMC7255347

[14] Channappanavar R, Fehr AR, Vijay R Dysregulated type I interferon and inflammatory monocyte–macrophage responses cause lethal pneumonia in SARS-CoV-infected mice. Cell Host Microbe 19(2), 181–193 (2016). 26867177 10.1016/j.chom.2016.01.007 PMC4752723

[15] Channappanavar R, Fehr AR, Zheng J IFN-I response timing relative to virus replication determines MERS coronavirus infection outcomes. J. Clin. Invest. 129(9), 3625–3639 (2019). 31355779 10.1172/JCI126363 PMC6715373

[16] Zhou Q, Chen V, Shannon CP Corrigendum: interferon-α2b treatment for COVID-19. Front. Immunol. 11, 615275 (2020). 33193462 10.3389/fimmu.2020.615275 PMC7653513

[17] Zuo Y, Liu Y, Zhong Q Lopinavir/ritonavir and interferon combination therapy may help shorten the duration of viral shedding in patients with COVID-19: a retrospective study in two designated hospitals in Anhui, China. J. Med. Virol. 92(11), 2666–2674 (2020). 32492211 10.1002/jmv.26127 PMC7300569

[18] Xie X, Jiang Y, Zeng Y, Liu H. Combination antiviral therapy with lopinavir/ritonavir, arbidol and interferon-α1b for COVID-19. Antivir. Ther. (2020) (Epub ahead of print). 10.3851/IMP3362 32496210

[19] Soy M, Keser G, Atagündüz P Cytokine storm in COVID-19: pathogenesis and overview of anti-inflammatory agents used in treatment. Clin. Rheumatol. 39(7), 2085–2094 (2020). 32474885 10.1007/s10067-020-05190-5 PMC7260446

[20] Jenkins MM, Mccaw TR, Goepfert PA. Mechanistic inferences from clinical reports of SARS-CoV-2. Infect. Dis. (Lond.) 52(8), 527–537 (2020). 32459123 10.1080/23744235.2020.1769853 PMC7265107

[21] Bost P, Giladi A, Liu Y Host-viral infection maps reveal signatures of severe COVID-19 patients. Cell 181(7), 1475–1488 (2020). 32479746 10.1016/j.cell.2020.05.006 PMC7205692

